# A case series of fatal meningoencephalitis in Mongolia: epidemiological and molecular characteristics of tick-borne encephalitis virus

**DOI:** 10.5365/wpsar.2018.9.1.003

**Published:** 2019-03-27

**Authors:** Uyanga Baasandavga, Burmaajav Badrakh, Natsagdorj Burged, Otgonsuren Davaajav, Tungalag Khurelsukh, Amber Barnes, Unursaikhan Ulaankhuu, Tsogbadrakh Nyamdorj

**Affiliations:** a National Center for Zoonotic Diseases, Ulaanbaatar, Mongolia.; b Mongolian Academy of Medical Science, Ulaanbaatar, Mongolia.; c Institute of Veterinary Medicine, Ulaanbaatar, Mongolia.; d Duke University, Durham, North Carolina, United States of America.

## Abstract

In Mongolia, the incidence and fatality rates of tick-borne encephalitis (TBE) have been increasing. We aimed to identify the epidemiological and molecular characteristics of tick-borne encephalitis virus (TBEV) associated with fatal meningoencephalitis in Mongolia.

We conducted a descriptive study of 14 fatal cases of TBE that occurred between 2008 and 2017 in Mongolia. Reverse transcription polymerase chain reaction (RT–PCR) was used to detect viral RNA in brain tissue. RT–PCR products from six patients who died from TBE between 2013 and 2017 were directly sequenced and analysed phylogenetically. Ticks collected from Selenge and Bulgan provinces were also tested for TBEV by RT–PCR.

Between 2008 and 2017, there were 14 fatal TBE cases in hospitals in Mongolia. The 14 patients who died reported receiving tick bites in Bulgan or Selenge province; 71.4% of deaths resulted from tick bites in Bulgan province. The TBE case fatality rate was 28.6% for patients in Bulgan province and 2.7% for those in Selenge province. All of the fatalities were men; the median age was 45 ± 12.6 years. Tick bites occurred between April and June in forested areas. In 2013, a 388 base pair fragment of the envelope (E) gene was obtained from a hospitalized patient. The closest relatives of this virus are Far-Eastern TBEV isolates.

The case fatality rate differed between two provinces where tick bites occurred. A higher number of TBE cases and the virulent Far-Eastern subtype occurred in patients in Bulgan province. This province should increase vaccination coverage, training, education and investigations.

## Introduction

Tick-borne encephalitis virus (TBEV) is a member of the genus *Flavivirus* of the Flaviviridae family. The virion consists of a single-stranded RNA molecule enclosed by the core membrane and the envelope (E) protein. The three genetically and antigenetically closely related TBEV subtypes (Western, Siberian and Far-Eastern) are not subject to significant antigenic variation. (*1*)

Tick-borne encephalitis (TBE) is a viral infectious disease that is transmitted by a bite from an infected tick and can progress to death. In Europe and Asia, between 10 000 and 15 000 TBE cases are reported annually. (*1*) Reported case fatality rates (CFR) differ based on virus subtypes: 20–40% for the Far-Eastern subtype, 6–8% for the Siberian subtype and 1–2% for the European subtype. (*2*)

TBEV can be transmitted to humans during the bite of several species of infected ticks, including *Ixodes scapularis*, *Ixodes ricinus* and *Ixodes persulcatus*; however, the main vector of TBEV is *Ixodes persulcatus*. (*2*) Researchers isolated TBEV from *Ixodes persulcatus* collected in Selenge and Bulgan provinces in the northern part of Mongolia. (*3*, *4*)

TBE has recently attracted attention because of the increasing incidence and consequent significant harm to humans. Since 2005, vaccination and educational campaigns have been administered throughout the affected areas of Mongolia, but human cases and fatalities from TBE continue to increase. The goal of this study was to identify the epidemiological and molecular characteristics of TBEV associated with fatal meningoencephalitis in Mongolia. Greater understanding of the virulence of TBEV in Mongolia and its distribution is urgently needed for the prevention of TBE.

## Methods

### Epidemiological characteristics of fatal cases

Data on fatal TBE cases were documented by the National Center for Zoonotic Diseases (NCZD), which has registered tick-borne diseases since 2005. (*5*) We used data of all cases that were confirmed at the National Reference Laboratory of NCZD by enzyme-linked immunosorbent assay (ELISA), immunofluorescence assay (IFA) and polymerase chain reaction (PCR). Confirmatory diagnostic definitions were:

- *Clinical criteria:* Patient with at least two of the following signs or symptoms without other known reasons: fever > 37.5 °C, headache, stiff neck, vomiting, paralysis or loss of consciousness.
- *Laboratory criteria*: IgM positive or IgG fourfold increase in pair serum OR detection of TBEV nucleic acid in any clinical specimen.
- *Exposure history:* Exposure is defined as a person who travelled or lived in forested areas with tick bites.
- Suspected cases: Patients with exposure history who fulfilled clinical criteria during the surveillance period.
- Confirmed cases: Suspected cases + laboratory confirmation.

A descriptive epidemiological study was conducted of 14 TBE patients who died in Mongolia between 2008 and 2017. All patients who died were bitten by ticks in areas of Bulgan and Selenge provinces. We collected clinical information on age, sex, occupation, province, clinical symptom onset dates, hospitalization dates and range of symptoms from the medical files. The CFR rate was calculated by the proportion of deaths among the laboratory-confirmed TBE cases in the province.

### Tick collection, processing and viral RNA extraction

A total of 65 ticks (*Ixodes persulcatus*) were collected by flagging in Selenge province (17 ticks) and Bulgan province (48 ticks) in 2017.

Ticks were collected from Bulgan and Selenge provinces in the areas the patients recalled being bitten by a tick. Ticks were sampled in July 2017 using flagging methods according to the guidance of NCZD. For tick collection, a tick drag method was conducted using a white cloth sized 60 × 100 cm. Ticks were stored alive in a 50 ml Falcon tube until they were investigated. Tick species were visually identified using a tick identification guide. (*6*)

Viral RNA was isolated from ticks using a Pure Link RNA Mini Kit (Thermo Fisher, Waltham, MA, USA) according to the manufacturer’s protocol. The ticks were frozen in liquid nitrogen then ground using a sterile mortar and pestle. The resulting homogenate was transferred to a 1.5 ml tube and mixed with 0.6 ml lysis buffer before viral RNA extraction.

### Post-mortem sample collection, processing and viral RNA extraction

Post-mortem tissue samples from seven of the TBE patients were stored at −70 °C in the NCZD laboratory; tissue samples of seven other deceased patients were not available because the families declined autopsies. We excluded one patient’s sample since results of the laboratory analysis of this sample were previously published in 2010. (*7*) Samples of cerebellum, cerebral cortex and spinal cord of the remaining six patients who died from TBE between 2013 and 2017 were selected.

Viral RNA was extracted from the supernatant of post-mortem nervous tissue using a Pure Link RNA Mini Kit (Thermo Fisher, Waltham, MA, USA) according to the manufacturer’s protocol. The extracted RNA was eluted from a spin cartridge column in a volume of 100 µL RNase-free water.

### RT–PCR with TBEV E gene specific primers

Reverse transcription (RT) was done using Super Script III (Thermo Fisher, USA) according to the manufacturer’s instruction. PCR was performed using taq-polymerase and sets of primers EncE-L (5′-GACCAGAGTGATCGAGGCTG-3′) and 1643-R (5′-GCCAGATCATTRAACCAGTC-3′), which flank the 388 bp fragment inside the E gene of the TBEV genome.

An RT–PCR master mixture was prepared using 2x Reaction mix 25 µl, Super Script®III (Thermo Fisher, USA) RT/Platinum® *Taq* Mix 2 µl, F and R primers each 1 µl, molecular water 19 µl and template RNA 2 µl (final volume 50 µ/sample; Super Script®III One-Step RT–PCR System with Platinum®Taq DNA Polymerase, #12574–026, (Thermo Fisher, USA). The RT–PCR conditions were: 1 cycle of 50 °C for 45 minutes and 94 °C for 5 minutes; 40 cycles of denaturation at 94 °C for 1 minute, annealing at 58 °C for 1 minute, and extension at 72 °C for 2 minutes; and final extension at 72 °C for 7 minutes. The PCR products were detected in UV light as ethidium bromide-stained 388-base pair bands electrophoresed with a marker on 1.5% agarose gel. A band detected on the gel was purified using the quick PCR Purification Kit (Qiagen, Hilden, Germany).

### Sequence analysis

Sequence analyses of the PCR products were conducted at the Institute of Veterinary Medicine of Mongolia using Genetic Analyser 3100 Xl with Bigdye v3.1 (Thermo Fisher, USA) according to the manufacturer’s protocol. Raw data sequences were analysed to create FASTA files with Sequence analyser v5.2 and Codoncode alignment v7.1. The sequence results were checked using the BLAST web site of the National Center for Biotechnology Information. (*8*) Phylogenic analyses of the PCR products sequences were performed using Crustal X v2.0 and MEGA 7.0.

### Ethical statement

Post-mortem tissue samples were collected and stored at the NCZD laboratory. Ethical clearance was not required as NCZD is responsible for analysis of zoonotic diseases.

## Results

### Epidemiological characteristics of fatal cases

**Fig. 1:**
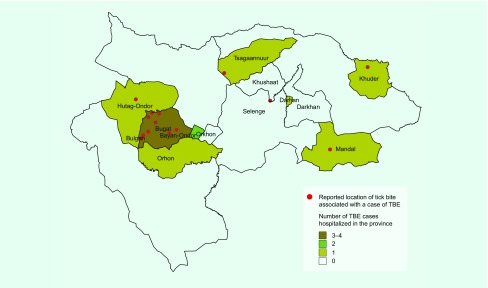
Distribution of 14 fatal TBE cases

Fourteen patient fatalities were registered in Mongolia between 2008 and 2017 for a CFR of 4.9% (14/287). Seven (50%) of the documented patients lived in Bulgan province, three (21.4%) in Selenge province, three (21.4%) in Orkhon province and one (7.1%) in Darkhan-Uul province (**Fig. 1**). By the tick bite area, total CFR was 28.6% (10/35) in Bulgan, and 2.7% (4/150) in Selenge province. The CFR range was 18.2–50% in Bulgan and 5.7–10% in Selenge province between 2008 and 2017.

Each red dot represents the location of a tick bite associated with a case of TBE. Provinces where patients with TBE were hospitalized are shaded in green and yellow.

All of the TBE fatalities were men who reported tick bites during the months of April through June (April: 1/14 [7.1%], May: 9/14 [64.3%], and June: 4/14 [28.6%]). The most common activities associated with tick bites were collecting plants (5/12, 41.6%), preparing wood (4/12, 33.3%), collecting animal horns (2/12, 16.7%) and herding livestock in forested areas (1/12, 8.3%). The median age of the fatal cases was 45 ± 12.6 years; the employments of the fatal cases included herder, driver, self-employed and unemployed (**Table 1**).

**Table 1:** Epidemiological characteristics of fatal TBE cases in Mongolia, 2008–2017

-	Characteristics	Number of fatalities (*n* = 14)	Percentage
**Sex**	Male	14	100
**Age group (years)**	30–39	6	42.9
	40–49	3	21.4
	50–59	2	14.3
	Above 60	3	21.4
**Employment**	Herder	5	35.7
	Unemployed	4	28.6
	Driver	2	14.3
	Private worker	2	14.3
	Pensioner	1	7.1
**Exposure month**	April	1	7.1
	May	9	64.3
	June	4	28.6
**Reason for tick bite**	Prepared wood	4	28.6
	Collected plants	5	35.7
	Herding livestock	1	7.1
	Collected horns	2	14.3
	Unknown	2	14.3
**Tick bite province**	Selenge	4	28.6
	Bulgan	10	71.4
**Diagnosis**	Meningoencephalitis	12	85.7
	Meningoencephalomyelitis	2	14.3

The median incubation period was 16 ± 11.4 days. Generally, fatalities occurred 8.1 ± 5.2 days after clinical symptoms developed. The median incubation period of fatal cases in Selenge province was 9 days with symptom onset dates from 1 May to 25 June. The median days between symptom onset and death was 10.7 days. The median incubation period of fatal cases in Bulgan province was 12.6 days with symptom onset dates between 24 May and 28 June. The median number of days between symptom onset and death was 6.7 days.

Patients with TBEV from a tick bite in Selenge province had a 1.4 times shorter incubation period than those in Bulgan province (*P* = 0.15). The number of days between symptom onset and death in Selenge province was 1.5 times longer than in Bulgan province (*P* < 0.05).

The most common clinical signs and symptoms were fever (12/14, [85.7%]); paralysis (12/14, [85.7%]); headache (11/14, [78.6%]); vomiting (10/14, [71.5%]); loss of consciousness (8/14, [71.5%]); stiff neck (8/14, [71.5%]); muscle ache (8/14, [71.5%]); and coxalgia, rash, blindness, and cough with bloody mucus (each reported by 1/14, [7.1%]) (**Table 2**). All patients who died were not vaccinated against TBE.

**Table 2:** Clinical symptoms of fatal TBE cases in Mongolia, 2008–2017

Province of tick bite	Age, sex	Incubation period (days)	Date of symptom onset	Initial symptoms reported	Date of hospitalization	Signs and symptoms recorded	Number of days between symptom onset and death
**Selenge**	38, M	6	22 Jun 2008	Headache	28 Jun 2008	Coma	8
	66, M	10	24 May 2015	Headache	24 May 2015	Loss of consciousness, coma, paralysis, coxalgia	23
	34, M	5	25 Jun 2015	Fever, headache, vomiting, stiff neck	28 Jun 2015	Loss of consciousness, coma, paralysis	8
	35, M	15	1 Jun 2017	Headache	4 Jun 2017	Loss of consciousness, coma, paralysis	4
**Bulgan**	50, M	8	20 June 2008	Fever, headache, vomiting, stiff neck	21 Jun 2008	Loss of consciousness, coma, paralysis, rash, cough with bloody mucus	12
	41, M	33	28 Jun 2013	Headache, vomiting	30 Jun 2013	Loss of consciousness, coma, paralysis, muscle ache	12
	46, M	4	24 May 2013	Fever, headache, vomiting	24 May 2013	Loss of consciousness, fever, coma, facial paralysis, blindness, muscle ache	5
	31, M	15	20 Jun 2013	Fever, headache, vomiting, stiff neck	22 Jun 2013	Loss of consciousness, paralysis, muscle ache	3
	43, M	14	10 Jun 2016	Fever, headache, vomiting	12 Jun 2016	Paralysis, muscle ache	4
	54, M	13	21 Jun 2016	Fever, headache, vomiting, stiff neck	23 Jun 2016	Paralysis, muscle ache	6
	34, M	Unknown	24 May 2017	Fever, vomiting, stiff neck	26 May 2017	Paralysis, muscle ache, heartache	5
	33, M	16	25 May 2017	Fever, headache, stiff neck	29 May 2017	Loss of consciousness, coma	11
	62, M	10	1 Jun 2017	Fever, headache, vomiting, stiff neck	3 Jun 2017	Paralysis, muscle ache	5
	60, M	38	31 May 2017	Fever, headache, vomiting, stiff neck	1 Jun 2017	Loss of consciousness, coma, paralysis, muscle ache	7

### Molecular epidemiology of fatal cases and infected ticks

TBEV RNA was detected in two ticks (11.7%) from Selenge province and six brain tissue samples from six patients who died (one in Selenge, five in Bulgan) between 2013 and 2017. By RT–PCR, the TBEV E gene was amplified from synthesized cDNA using primer sets targeting the E gene of TBEV that produced a 388 base pair fragment. This fragment of E gene was isolated from six patients who died and two ticks. No nucleotide structures suitable for sequence analysis could be identified in either tick sample.

One fatal case from Bulgan province in 2013 had an identifiable nucleotide structure. Samples from the other five fatalities did not yield enough product for sequencing. The nucleotide sequence of the sample was most similar (90%) to the isolate TBEV-MN-2008 (HM133639.1) according to GenBank data. (*8*) Phylogenetic analysis showed that the isolated virus belonged to the Far-Eastern subtype (strains of the 886 and X1subtype) of TBEV. (*7*, *9*, *10*)

## Discussion

In our study, all of the TBE fatalities were men who reported tick bites during the months of April through June. In other studies, men are more affected than women by fatal TBE. (*11*, *12*) The seasonal distribution of TBE cases depends on the activity of the tick species; *Ixodes persulcatus* generally emerge between April and May, but *Ixodes ricinus* activity periods may occur in April, May, October and November. (*13*) Our study shows, *Ixodes persulcatus* becoming important vector of TBE in forested areas of Selenge and Bulgan provinces of Mongolia.

The CFR for TBE differs by virus subtype, including less than 2% for the European subtype, 20–40% for the Far-Eastern subtype, and 6–8% in the Siberian subtype. (*2*) In our study, the CFR range in Bulgan province was 18.2–50%, similar to that of the Far-Eastern subtype, and the CFR range in Selenge province was 5.7–10%, similar to that of the Siberian subtype. In previous studies of TBEV subtypes in Mongolia, Siberian subtype was found in *Ixodes persulcatus* of Selenge and Bulgan provinces. (*7*, *14*) Our study indicates that the Far-Eastern subtype is also present in humans in Bulgan province. This is the second confirmed case of lethal TBE caused by the Far-Eastern subtype in Bulgan province, but there has been no evidence of the Far-Eastern subtype found in ticks from Bulgan province. Despite the fatalities in northern Mongolia, few public health officials recommend expanding TBE vaccination for Selenge and Bulgan provinces. (*3*) Most studies, vaccination efforts and TBE trainings have focused primarily in Selenge province which has the largest population of *Ixodes persulcatus*. (*3*, *4*)

We found that the Far-Eastern subtype of TBEV is the predominant virus among the fatal TBE cases in Bulgan province. Therefore, this province must increase vaccination coverage, training and education; also, it needs to conduct further comprehensive investigations in the epidemiology of TBE. More research is necessary to understand why the Far-Eastern subtype has not yet been found in *Ixodes persulcatus* collected in the province and whether a different species may be contributing to human disease.

This study has some limitations. The patient sample size was small, and half of the tissue samples could not be analysed. Finally, more tick samples are necessary to fully identify the subtypes circulating in ticks from these regions.

## Conclusion

Despite these drawbacks, this study provides important epidemiological and molecular analysis of recent TBE cases and associated fatalities due to meningoencephalitis. As this tick-borne disease continues to be a public health concern to endemic provinces in Mongolia, our study can help prevent infection and subsequent serious or fatal illness. These findings support expanded vaccinations for Bulgan province and continued vaccination in Selenge province. At-risk individuals from both provinces should be targeted for education and prevention messages. More research is necessary to discover which subtypes of TBEV are circulating among tick vectors in these regions and how these subtypes may impact disease susceptibility and recovery in patients. A more coordinated effort is needed between health research and public policy officials to combat the increasing risk of TBEV transmission in Mongolia.

## References

[1] Süss J. Tick-borne encephalitis in Europe and beyond–the epidemiological situation as of 2007. Euro Surveill. 2008 6 26;13(26):18916. 18761916

[2] Gritsun TS, Lashkevich VA, Gould EA. Tick-borne encephalitis. Antiviral Res. 2003 1;57(1-2):129–46. 10.1016/S0166-3542(02)00206-1 12615309

[3] Khasnatinova MA, Tserennorov D, Nyamdavaa P, Glushenkova T, Arbatskaya E, Bataa J, et al. Tick-borne encephalitis virus in Mongolia. Int J Infect Dis. 2010;14:372–3. 10.1016/j.ijid.2010.02.449

[4] Boldbaatar B, Jiang RR, von Fricken ME, Lkhagvatseren S, Nymadawa P, Baigalmaa B, et al. Distribution and molecular characteristics of rickettsiae found in ticks across Central Mongolia. Parasit Vectors. 2017 2 2;10(1):61. 10.1186/s13071-017-1981-3 28153052 PMC5289011

[5] The history of NCZD establishment. Ulaanbaatar: National Center for Zoonotic Disease; 2018 (https://nczd.gov.mn/?page_id=9295&lang=en)

[6] Hoskins JD. Ixodid and argasid ticks. Keys to their identification. Vet Clin North Am Small Anim Pract. 1991 1;21(1):185–97. 10.1016/S0195-5616(91)50018-8 2014622

[7] Khasnatinov MA, Danchinova GA, Kulakova NV, Tungalag K, Arbatskaia EV, Mironova LV, et al. [Genetic characteristics of the causative agent of tick-borne encephalitis in Mongolia]. Vopr Virusol. 2010 May-Jun;55(3):27–32. [in Russian] 20608078

[8] Basic local alignment search tool. Bethesda, MD: National Center for Biotechnology Information; 2018 (http://www.ncbi.nlm.nih.gov/BLAST/)

[9] Tkachev SE, Demina TV, Dzhioev Yu P, Kozlova IV, Verkhozina MM, Doroshchenko EK, et al. Genetic studies of tick-borne encephalitis virus strains from western and eastern Siberia. In: Flavivirus Encephalitis. InTechOpen. 2011;12(3):235–54.

[10] Bertrand YJK, Johansson M, Norberg P. Revisiting recombination signal in the tick-borne encephalitis virus: a simulation approach. PLoS One. 2016 10 19;11(10):e0164435. 10.1371/journal.pone.0164435 27760182 PMC5070875

[11] Tajima Y, Yaguchi H, Mito Y. Fatal meningoencephalomyetis due to the tick-borne encephalitis virus: the first detailed neurological observation in a Japanese patient from the central part of Hokkaido Island. Intern Med. 2018;57(6):873–6. 10.2169/internalmedicine.8437-16 29540659 PMC5891530

[12] Kuivanen S, Smura T, Rantanen K, Kämppi L, Kantonen J, Kero M, et al. Fatal tick-borne encephalitis virus infections caused by Siberian and European subtypes, Finland, 2015. Emerg Infect Dis. 2018 5;24(5):946–8. 10.3201/eid2405.171986 29664395 PMC5938788

[13] Korenberg EI. Seasonal population dynamics of ixodes ticks and tick-borne encephalitis virus. Exp Appl Acarol. 2000;24(9):665–81. 10.1023/A:1010798518261 11227825

[14] Frey S, Mossbrugger I, Altantuul D, Battsetseg J, Davaadorj R, Tserennorov D, et al. Isolation, preliminary characterization, and full-genome analyses of tick-borne encephalitis virus from Mongolia. Virus Genes. 2012 12;45(3):413–25. 10.1007/s11262-012-0795-9 22847274

